# Parenting behaviour and paranoia: a network analysis and results from the National Comorbidity Survey-Adolescents (NCS-A)

**DOI:** 10.1007/s00127-020-01933-6

**Published:** 2020-08-18

**Authors:** Poppy Brown, Felicity Waite, Daniel Freeman

**Affiliations:** 1 Department of Psychiatry, Warneford Hospital, University of Oxford, Warneford Lane, Oxford, OX3 7JX UK; 2 Oxford Health NHS Foundation Trust, Oxford, UK

**Keywords:** Paranoia, Delusions, Parental abuse, Parental over-protectiveness, Parental care

## Abstract

**Purpose:**

Parenting behaviours—including the extent to which parents are protective, hostile, or caring—likely impacts whether a child develops a sense of vulnerability that carries forward into adulthood. Ideas of vulnerability are a contributory factor to the occurrence of paranoia. Our aim was to assess whether there is an association between specific parenting behaviours and paranoia.

**Method:**

We examined cross-sectional associations of parenting and paranoia in an epidemiologically representative cohort of 10,148 adolescents (National Comorbidity Survey-Adolescents; NCS-A) and a second dataset of 1286 adults in Oxfordshire. Further, a network analysis was conducted with paranoia, parenting behaviours, and cognitive-affective variables (compassion, self-esteem, anxiety, and depression). Overprotectiveness, verbal abuse, physical abuse, and amount of care were assessed in mothers and fathers separately.

**Results:**

Nearly all parenting variables were significantly associated with paranoia, with parental verbal and physical abuse showing the largest associations. For example, the odds of reporting paranoia was over four times higher for those in the adult sample reporting a lot of paternal verbal abuse, compared to those reporting none (OR = 4.12, *p* < 0.001, CI 2.47–6.85). Network analyses revealed high interconnectivity between paranoia, parenting behaviours, and cognitive-affective variables. Of the parenting variables, paranoia most strongly interacted with paternal abuse and maternal lack of care.

**Conclusion:**

There are associations between participants’ self-reported experiences of parental behaviours and paranoia. Despite being associated with paranoia, cognitive-affective variables did not appear to mediate the relationship between parenting and paranoia, which is surprising. What might explain the link therefore remains to be determined.

## Introduction

Paranoia exists on a spectrum of severity in the general population: many people have a few paranoid thoughts and a few people have many [1–3]. It is therefore possible to learn about the clinical disorder by studying milder variants in the general population. A contributory causal factor in the occurrence of paranoia is negative beliefs about the self [4]. Negative views of the self-engender a sense of vulnerability that paranoia builds upon. How do these negative views of the self-develop? The influence of the environment on the occurrence of paranoia has been found to be substantial; with non-shared environmental influences on paranoia estimated to be 0.49 [5]. An obvious potential contributory factor that could affect views of the self is parenting behaviour. This paper investigates for the first time the association between specific aspects of parenting, cognitive-affective processes, and paranoia.

In a theoretical model, it is hypothesised that negative self-beliefs lead to feeling inferior, apart, and vulnerable, and that paranoia builds upon these concerns [4]. Negative beliefs about the self (e.g., that the self is vulnerable) are correlated with both clinical and non-clinical levels of paranoia [6–9]. Experimental studies in non-clinical samples have shown that increasing or decreasing negative self-beliefs affects the occurrence of paranoia [10, 11]. Furthermore, it has been shown that treating negative self-beliefs in patients with persecutory delusions can lead to a reduction in paranoia [12]. Negative self-beliefs are also strongly associated with a lack of self-compassion [13]. Experimental manipulations have found that self-compassion interventions can reduce both negative beliefs [14, 15], and paranoia in non-clinical [16, 17] and clinical samples [18]. Compassionate interventions focused on beliefs about others also show promise. Negative beliefs about others (e.g., that others are devious) have been correlated with clinical and non-clinical levels of paranoia [6]. Brown et al. showed that an intervention that trained compassion for others resulted in a reduction in paranoia. The empirical literature shows a tight connection between beliefs about the self and others and the occurrence of paranoia.

How might the beliefs about the self and others form in the first place? Childhood physical abuse, sexual abuse, and other victimisation experiences have been associated with paranoia and may plausibly partly exert influence via beliefs about the self and others [19, 20]. Parenting behaviour is also a plausible contributory factor to the development of negative beliefs. Parental behaviours have been investigated in relation to a number of mental health conditions. For example, over-protectiveness and low parental care have been associated with anxiety [21, 22] and depression [23]. Such parental behaviours have also been associated with schizophrenia. Read et al. review a number of studies investigating ‘affectionless control’, that is, high perceived overprotectiveness but low care by parents of individuals with schizophrenia [24]. They found evidence for an association between affectionless control and schizophrenia, particularly among fathers. Parker et al. suggest that levels of parental protectiveness can range from excessive contact, intrusion, control, infantilisation, and the prevention of independent behaviour, to allowing of complete autonomy and independence [25]. Similarly, levels of care can range from emotional warmth, affection, closeness, and empathy, to emotional coldness, neglect, and indifference. While it might be argued that the link between this kind of parental behaviour and schizophrenia is predominately genetic, Onstad et al. showed that for both monozygotic (MZ) and dizygotic (DZ) twins, the twin later diagnosed with schizophrenia reported more overprotection than the other twin [26]. Given MZ twins are genetically identical, this suggests the association between parental over-protection and schizophrenia symptoms is not purely a genetic one.

How might parenting behaviours link to paranoia? Perhaps the experience of overprotection could lead a child to develop schemas about the world as dangerous and themselves as vulnerable, to explain the protection. Similarly, experiencing low care from parents could lead a child to develop the kinds of negative self-beliefs that paranoia builds upon, e.g., that they are unworthy of care and therefore inferior to others. Finally, negative beliefs about the self and others are often developed in the context of adverse interpersonal experiences [4]. Experiencing abuse, particularly from a trusted figure such as a parent during childhood, could therefore also result in the development of negative schemeas about the self and others. Indeed Read and Gumley argue that maltreatment by attachment figures can lead to a disorganised attachment style, which reflects a combination of negative beliefs about the self and others [24]. They suggest these beliefs are then carried forward into adulthood and can contribute to the onset of psychosis. While paranoia has been shown to be associated with having an insecure attachment style [27], particularly a disorganised insecure attachment [28], and with experiencing abuse or being taken into institutional care [29], its association with these more specific parental behaviours has not been determined.

In this paper, we examine associations in two samples (a national epidemiological group and a newly recruited sample of adults) between parenting, cognitive affective processes, and paranoia. The aim was to use the first sample as an initial test of the model, with the second sample used to explore the relationships in greater detail with more robust measures included. We also make use of a network analysis to conceptualise the interplay between these variables [30, 31]. Network analysis statistically estimates complex interactions thereby allowing visualisation of the strength of associations between groups of variables, while also giving insight into potential causal processes [32]. The visualisation of such complex interplay enables greater learning from cross-sectional data, and the drawing of potential causal pathways helps to generate hypotheses for future research [33]. More generally, the network approach is increasingly seen as an important method for allowing psychological processes to be analysed as products of complex and dynamic systems [34].

Our hypotheses were as follows. First, that regression analysis would show positive associations between maternal and paternal overprotectiveness and paranoia and between maternal and paternal abuse and paranoia, and negative associations between amount of maternal and paternal care and paranoia in both participant groups. Second, these associations would be apparent when analysing variables as part of a network. Third, within the network, cognitive-affective variables such as levels of anxiety and self-esteem would provide a mediating pathway between paranoia and parenting behaviours.

## Method

Associations between parenting behaviour and paranoia were first tested in the National Comorbidity Survey Replication Adolescent Supplement (NCS-A) [35] and then in a new survey conducted to assess the key variables in greater depth. The NCS-A survey was administered using computer assisted, face-to-face, individual interview by professional interviewers employed by the Survey Research Centre. The interview schedule was based on the World Health Organisation Composite International Diagnostic Interview (WMH-CIDI). Merikangas et al. report further details of the adaptions to measures in the NCS-A [36]. A hard copy of the instrument is posted at www.hcp.med.harvard.edu.ncs. The new survey was administered via Qualtrics, an online questionnaire platform.

### Participants

#### NCS-A

The NCS-A sample included 10,148 adolescents aged 13–17 years old. 9244 adolescent students were selected from a representative sample of 320 schools in the same nationally representative sample as the National Comorbidity Survey-Replication (NCS-R) (response rate 74.7%). The remaining 904 participants were from the same households of those that took part in the National Comorbidity Survey-Replication (response rate 85.9%). The mean age was 15.18 years (SD = 1.51) and 48.9% (*n* = 4962) of the sample were male, 51.1% (*n* = 5186) female.

#### Oxfordshire participant group

The second participant group consisted of 1231 adults (aged 18 or over). Participants took part in the survey as part of the screening process for an experimental study that was advertised via social media adverts in the region of Oxfordshire, UK**.** The mean age of this survey group was 41.54 years (SD = 15.95). Data on participant gender were not collected for the first 207 participants. Of the remaining 1024 participants, 23.7% (*n* = 243) were male and 76.3% (*n* = 781) female. It is typical for online surveys to receive a considerably higher response rate from women [37, 38]

### Measures

#### NCS-A

##### Paranoia

Participants were asked to respond to the following statement with ‘true’, ‘false’, or ‘do not know’: ‘People often make fun of me behind my back’. This item has previously been used as a brief measure of paranoia [39]. A correlation difference test supported the internal validity of the measure by showing that this single-item measure of paranoia (*n* = 857) had a significantly higher correlation with a 16-item measure of paranoia (the Green et al. Paranoid Thoughts Scale-Part B, Green et al., 2008) [40] (*r* = 0.56), than with a measure anxiety (*r* = 0.38), *z* = 15.00, *p* < 0.0001.

##### Parental behaviour

Participants were asked to respond to the following statements with ‘a lot’, ‘some’, ‘a little’, or ‘not at all’ for both mother and father figures separately: ‘How much did he/she really care about you?’; ‘How overprotective was he/she?’. Participants were asked to respond to the following lists and statements with ‘often’, ‘sometimes’, ‘not very often’, or ‘never’ for both mother and father figures separately: ‘When you were growing up, how often did he/she do any of these things to you?’: ‘insulted or swore, shouted, yelled or screamed, threatened to hit’ [verbal abuse (List A)]; ‘pushed, grabbed or shoved, threw something, slapped or hit’ [physical abuse (List B)]; ‘kicked, bit or hit with a fist, beat up, choked, burned or scalded, threatened with a knife or gun’ [severe physical abuse (List C)].

#### Oxfordshire participant group

The Oxfordshire participant group completed the same measures of paranoia and parental behaviour described for the NSC-A dataset, as well as the following measures:

##### Paranoia

Participants completed the Green et al. Paranoid Thoughts Scale—Part B (GPTS-B) [40]. This is a 16-item scale assessing ideas of persecution over the past month such as ‘I was convinced there was a conspiracy against me’ and ‘I was sure someone wanted to hurt me’ on a 1–5 scale (1 = not at all, 5 = totally). Scores can range from 16 to 80; higher scores reflect greater paranoia. The scale is well validated for use in both clinical and non-clinical samples [41] and has strong concurrent validity with paranoia severity as assessed by clinical interviews and by controlled virtual reality tests [42, 43]. Using item response theory analysis with over 10,000 individuals, the GPTS-B has been shown to demonstrate high reliability (*a* > 0.95) across both mild and severe ends of the paranoia spectrum [44]. Test–retest reliability has also been shown to be good, with an intra-class correlation coefficient of 0.81 [40].

##### Parenting

The Measure of Parenting Style (MOPS) [45] was used. This contains 15 items measuring specific maternal parenting behaviours and the same 15 items measuring paternal parenting behaviours. It was developed to overcome shortcomings of the Parental Bond Instrument [25] and assesses reported parental indifference, abuse, and over-control separately for mothers and fathers. Higher scores reflect higher reported levels of each behavior. Alpha coefficients of internal consistency for each of the six subscales range from 0.76 to 0.93 [45].

Although two of the subscales were named differently from the parenting questions included in the NCS-A dataset (indifference vs. amount of care, and over-control vs. over-protection), they were taken in our study to be measuring the same constructs. This was justified upon Parker et al.’s descriptions of both over-protection and care described above [45]. The abuse items in the MOPS were similar to those in the NCS-A dataset in separately measuring both physical and verbal abuse.

##### Self-compassion

The self-compassion scale-short form (SCS-SF) was used [46]. The scale consists of 12 items asking about how respondents typically act towards themselves in difficult times, rated on a Likert scale of one (almost never) to five (almost always), meaning higher scores reflect higher levels of self-compassion. There are six subscales, but use of a total score is recommended when using the short form. The SCS-SF demonstrates good internal consistency (Cronbach’s *α*  > 0.85 and a near-perfect correlation with the long form of the scale when using total scores (*r* > 0.96) [46].

##### Compassion for others

Participants were given the Compassion Scale [47], a 24-item scale measuring how respondents typically act towards others. As with the SCS-SF, items are rated on a Likert scale of one (almost never) to five (almost always) and there are six subscales, but a total score can also be used. Higher scores reflect higher levels of compassion for others. The scale demonstrates good internal consistency (Cronbach’s *α*  = 0.9) [47].

##### Anxiety and depression

The Patient Health Questionnaire-4 (PHQ-4) [48] is a brief four-item scale for anxiety and depression that has been well validated for detection of anxiety and depression in clinical samples [49]. Two items measure anxiety over the past two weeks and two measure depression over the past two weeks. Higher scores reflect greater anxiety and depression. Internal consistency for the scale is good (Cronbach’s *α*  = 0.85) [48]. The two item measure of anxiety used has shown high sensitivity for identifying generalised anxiety (88%), panic (76%), and social anxiety (70%), as well as moderate sensitivity for PTSD (59%) [50].

##### Self-esteem

The Rosenberg Self-Esteem Scale [51] is a highly used ten-item measure of global self-worth that measures positive and negative feelings about the self. Items are answered using a four-point Likert scale ranging from strongly agree to strongly disagree. Scores range from 10 to 30. Five items are reversed scored so that higher total scores indicate higher self-esteem.

### Analysis

#### NCS-A data

The NCS-A data were analysed using the Statistical Package for the Social Sciences [52]. The data were weighted to adjust for within-household differential probabilities of respondent selection. Details of the rationale and process of weighting have previously been reported [35, 53]. Logistic regressions were used to test the associations between the assessments of parental behaviour and paranoia. Standard mediation analyses were not conducted due to the cross-sectional nature of the data [54]. Gender was included as a co-variate in all analyses. All tests were two-tailed. The primary analysis was conducted separately for mother and father figures, given that interactions between them would be based on small amounts of data for key categories.

#### Oxfordshire data

First, identical logistic regressions as above were conducted using the same measures of parenting and paranoia as were included in the NSC-A dataset. Second, simple regressions were conducted for the more in-depth measures of parenting and paranoia completed by the Oxfordshire participant group.

Network analysis with the measures from the Oxfordshire survey was conducted in R, version 3.6.1 [55]. A network modelling approach was used to estimate the partial correlations between paranoia and the other measures. In network analysis, variables are represented by nodes. Two nodes may be connected by an edge. Edges represent an association between two variables after controlling for all other variables included in the network, i.e., a partial correlation. The absence of an edge between two variables indicates that the partial correlation is zero after controlling for all other variables, known as conditional independence. Associations are visualised in a network where the thickness and saturation of the edge colour corresponds to the strength of the relationship [56].

Using the package qgraph, a Gaussian graphical model was fitted [56]. A regularisation technique with the Least Absolute Shrinkage and Selection Operator (LASSO) was used to overcome any potential sampling variation and limit the estimation of false positives [57]. The LASSO regularisation shrinks estimates by employing a penalty that limits the sum of the partial correlation coefficients [58]. The degree of regularisation is controlled by a tuning parameter, which is selected to optimise the model fit by minimising the Extended Bayesian Information Criterion (EBIC) [59]. The EBIC hyperparameter is set between 0 and 0.5, with a lower parameter resulting in more potential false edges being retained, and a higher parameter potentially omitting true edges from the network [58]. A hyperparameter of 0.3 was therefore chosen. Using the package bootnet, a non-parametric bootstrap with 5000 interactions was conducted, to construct 95% confidence intervals for each edge [30]. Due to the method of regularisation edge weights are biased towards zero. Consequently, reported confidence intervals cannot be interpreted as a significance test against zero [30].

Two separate network models were constructed to show the shortest path between paranoia and every other variable, and between the parenting variable found to have the strongest edge with paranoia and every other variable using Dijkstra’s algorithm [60]. The shortest path represents the quickest route for an interaction to occur between two variables, calculated using the strength of edge weights along each potential route. In this way, even though two nodes may share a direct path, an indirect route via an intermediary node may consist of stronger associations and therefore be a quicker route. Redundant edges are then supressed. Such a network is helpful for highlighting likely mediation pathways.

## Results

Twenty-three per cent (*n* = 2302) of participants in the NCS-A participant group endorsed the paranoia item “People often make fun of me behind my back”. In the Oxfordshire group, 18% (n = 226) endorsed the paranoia item. Table 1 summarises the results of the logistic regressions for both participant groups. Odds ratios of above 1.0 indicate a positive association, whereas odds ratios of below 1.0 indicate a negative association.

**Table 1:** The cross-sectional relationship between parental behaviours and paranoia, controlling for gender

	NCS-A Sample			Oxfordshire Sample				
	*N* (no. who endorsed paranoia item/no. who did not endorse paranoia item/% who endorsed paranoia item)	Odds ratio	*p* value	95% CI	*N* (no. who endorsed paranoia item / no. who did not endorse paranoia item / % who endorsed paranoia item)	Odds ratio	*p* value	95% CI
Overprotectiveness								
Mother figure								
A lot	2962 (852, 2110, 28.8)	1.62	< 0.001**	1.31–1.99	229 (39, 190, 17.0)	0.674	0.095	0.43–1.07
Some	2834 (559, 2275, 19.7)	1.12	0.310	0.90–1.38	282 (45, 237, 16.0)	0.626	0.039*	0.40–0.98
A little	2387 (408, 1979, 17.1)	0.95	0.664	0.77–1.18	194 (31, 163, 16.0)	0.615	0.050*	0.38–1.00
Not at all	893 (163, 730, 18.3)				257 (62, 195, 24.1)			
Father figure								
A lot	2512 (720, 1792, 28.7)	1.31	0.002**	1.12–1.55	132 (23, 109, 17.4)	0.872	0.616	0.51–1.49
Some	2362 (456, 1906, 19.3)	0.89	0.180	0.75–1.06	214 (37, 177, 15.3)	0.896	0.629	0.58–1.40
A little	2565 (477, 2088, 18.6)	0.93	0.383	0.79–1.10	228 (38, 190, 16.7)	0.854	0.479	0.55–1.32
Not at all	1637 (329, 1308, 20.1)				388 (79, 309, 20.4)			
Verbal abuse (List A)								
Mother figure								
Often	363 (128, 235, 35.3)	2.17	< 0.001**	1.70–2.78	172 (54, 188, 31.4)	2.57	0.001**	1.51–4.39
Sometimes	1262 (376, 886, 29.8)	1.79	< 0.001**	1.51–2.11	222 (56, 166, 25.2)	1.82	0.026*	1.07–3.08
Not very often	2432 (544, 1888, 22.4)	1.38	< 0.001**	1.19–1.59	310 (38, 272, 12.3)	0.97	0.911	0.57–1.66
Never	5044 (939, 5044, 18.6)				258 (29, 229, 11.2)			
Father figure								
Often	302 (109, 193, 36.1)	1.50	< 0.001**	1.15–1.96	126 (52, 74, 41.3)	4.12	< 0.001**	2.47–6.85
Sometimes	1230 (360, 870, 29.3)	1.19	0.003**	1.01–1.40	198 (45, 153, 22.7)	2.03		1.25–3.30
Not very often	2075 (407, 1668, 19.6)	0.76	0.041*	0.66–0.88	272 (39, 233, 14.3)	1.29	0.004**	0.79–2.09
Never	5494 (1111, 4383, 20.2)				366 (41, 325, 11.2)		0.303	
Physical abuse (List B)								
Mother figure								
Often	99 (44, 55, 44.4)	2.61	< 0.001**	1.72–3.96	87 (23, 64, 26.4)	1.93	0.028*	1.07–3.47
Sometimes	392 (129, 263, 32.9)	1.71	< 0.001**	1.35–2.15	154 (49, 105, 31.8)	2.47	< 0.001**	1.53–3.98
Not very often	877 (236, 641, 26.9)	1.34	0.001**	1.12–1.60	278 (50, 228, 18.0)	1.34	0.183	0..87–2.06
Never	7734 (1578, 6156, 20.4)				443 (55, 388, 12.4)			
Father figure								
Often	97 (41, 56, 42.3)	1.98	0.002**	1.29–3.03	64 (24, 40, 37.5)	2.77	0.001**	1.52–5.02
Sometimes	338 (107, 231, 31.7)	1.36	0.018*	1.05–1.76	137 (39, 98, 28.5)	1.98	0.005**	1.23–3.17
Not very often	632 (163, 469, 25.8)	1.08	0.451	0.88–1.33	223 (42, 181, 18.8)	1.37	0.152	0.89–2.10
Never	8035 (1676, 6359, 20.9)				538 (72, 466, 13.4)			
Severe physical abuse (List C)								
Mother figure								
Often	23 (11, 12, 47.8)	3.18	0.006**	1.39–7.30	14 (5, 9, 35.7)	1.89	0.293	0.58–6.18
Sometimes	39 (12, 27, 30.8)	1.56	0.212	0.78–3.12	19 (4, 15, 21.1)	0.80	0.705	0.244–2.60
Not very often	108 (39, 69, 36.1)	1.91	0.002**	1.27–2.89	42 (16, 26, 38.1)	2.35	0.014*	1.19–4.64
Never	8933 (1926, 7007, 21.6)				887 (152, 735, 17.1)			
Father figure								
Often	27 (8, 19, 29.6)	1.13	0.779	0.48–2.67	15 (5, 10, 33.3)	2.20	0.178	0.70–6.93
Sometimes	58 (15, 43, 25.9)	1.04	0.904	0.57–1.91	34 (15, 19, 44.1)	3.97	< 0.001**	1.92–8.19
Not very often	100 (32, 68, 32.0)	1.41	0.124	0.91–2.19	55 (23, 32, 41.8)	3.69		2.08–6.55
Never	8918 (1933, 6985, 21.7)				858 (134, 724, 15.6)		< 0.001**	
How much care								
Mother figure								
A lot	8757 (1879, 6878, 21.5)	0.52	0.190	0.20–1.38	635 (81, 554, 12.8)	0.25	0.001**	0.12–0.56
Some	192 (58, 134, 30.2)	0.69	0.471	0.25–1.90	200 (51, 149, 25.5)	0.54	0.126	0.24–1.19
A little	51 (14, 37, 27.5)	0.56	0.313	0.18–1.74	95 (31, 64, 32.6)	0.64	0.304	0.28–1.49
Not at all	18 (7, 11, 38.9)				32 (14, 18, 43.8)			
Father figure								
A lot	8217 (1719, 6498, 20.9)	0.47	0.001**	0.30–0.72	574 (82, 492, 14.3)	0.39	0.002**	0.22–0.70
Some	531 (149, 382, 28.1)	0.67	0.094	0.42–1.07	216 (40, 176, 18.5)	0.41	0.005**	0.22–0.77
A little	177 (55, 122, 31.1)	0.77	0.324	0.45–1.30	102 (25, 77, 24.5)	0.51	0.044*	0.26–0.98
Not at all	93 (35, 58, 37.6)				70 (30, 40, 42.9)			

Dependent variable: ‘People often make fun of me behind my back’

Reference group: ‘Not at all’/‘Never’

*Significant at *p* < 0.05

**Significant at *p* < 0.01

In the NCS-A participant group, reporting ‘a lot’ of maternal or paternal overprotectiveness was significantly associated with having a higher likelihood of reporting paranoia. For example, the odds of reporting paranoia was 1.62 times higher for those who reported ‘a lot’ of overprotectiveness from their mother figure, compared with those who reported ‘none’. Conversely, in the Oxfordshire participant group, the odds ratios were in the opposite direction suggesting a negative association between reporting overprotectiveness and reporting paranoia. However, in only one instance did this reach statistical significance, and the confidence intervals for these results were also wide and mostly crossing 1.0. Patterns for all other variables across the two samples were consistent. Reporting verbal abuse and physical abuse were associated with a higher likelihood of reporting paranoia, and reporting a lot of care was conversely associated with a low likelihood of reporting paranoia.

### Oxfordshire sample regressions

Table 2 displays the results of the regressions. The GPTS-B was significantly positively correlated with all subscales of the MOPS indicating that higher levels of parental indifference, control and abuse were associated with greater endorsement of paranoid thoughts. Anxiety and depression were also significantly positively correlated with paranoia, whereas higher levels of self-compassion, compassion for others, and self-esteem were significantly negatively correlated with paranoia.

**Table 2:** Correlations between GPTS-B and all other measured variables

	*n*	Correlation with GPTS-B (Pearson)	*p* value
Mother indifference	1252	0.298	< 0.001
Mother control	1252	0.302	< 0.001
Mother abuse	1252	0.270	< 0.001
Father indifference	1174	0.280	< 0.001
Father control	1174	0.287	< 0.001
Father abuse	1174	0.264	< 0.001
Self-compassion	867	− 0.407	< 0.001
Compassion for others	867	− 0.226	< 0.001
Self-esteem	866	− 0.435	< 0.001
Anxiety	867	0.473	< 0.001
Depression	867	0.482	< 0.001

### Network analysis

**Fig. 1:**
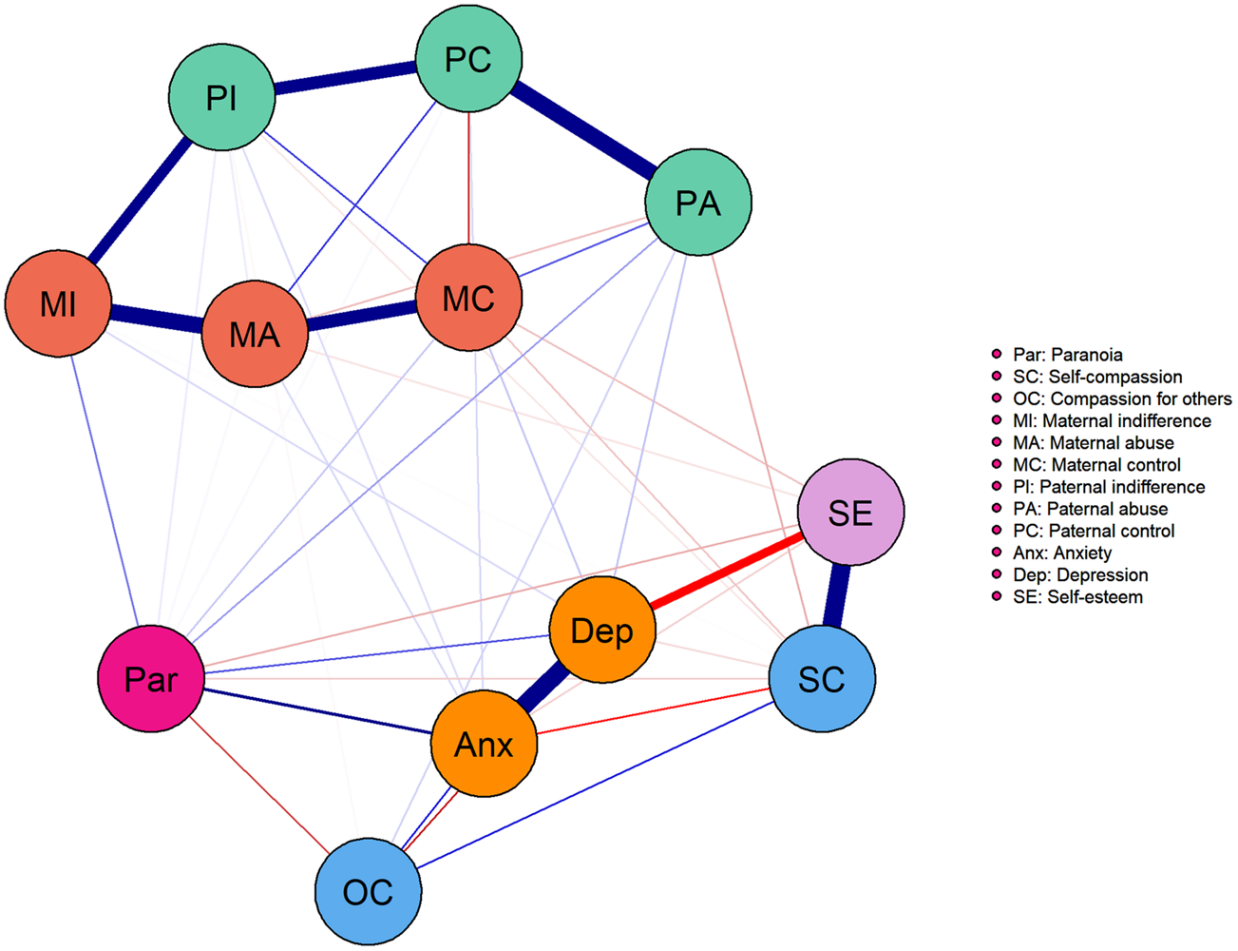
Fully estimated network. Blue lines indicate positive associations; red indicates negative association. Line thickness and colour saturation correspond to strength of relationship

Figure 1 shows the fully estimated network. Table 3 displays edge weights from paranoia to all other variables and their 95% confidence intervals. The network is highly interconnected within and between the parenting variables, cognitive-affective variables, and paranoia, confirming the presence of the significant associations seen in the regression results. Paranoia was most significantly associated with anxiety, with slightly smaller associations to all of the other cognitive-affective variables. The largest edges between paranoia and parenting behaviours were between paranoia and maternal indifference, and between paranoia and paternal abuse. A slightly smaller edge was present between paranoia and maternal control, with only very weak edges between paranoia and paternal indifference, paternal control, and maternal abuse. The strongest edge between the parenting variables and the cognitive-affective variables was between maternal control and self-compassion.

**Table 3:** Edge weights and confidence intervals between paranoia and all other variables

	Edge weight to paranoia (*r*)	95% confidence interval
Mother indifference	0.08	0.01; 0.15
Mother control	0.03	− 0.03; 0.09
Mother abuse	0.00	− 0.04; 0.05
Father indifference	0.01	− 0.03; 0.05
Father control	0.00	− 0.04; 0.05
Father abuse	0.06	− 0.01; 0.12
Self-compassion	− 0.03	− 0.08; 0.03
Compassion for others	− 0.11	− 0.19; − 0.03
Self-esteem	− 0.05	− 0.11; 0.01
Anxiety	0.19	0.11; 0.26
Depression	0.10	0.03; 0.17

**Fig. 2:**
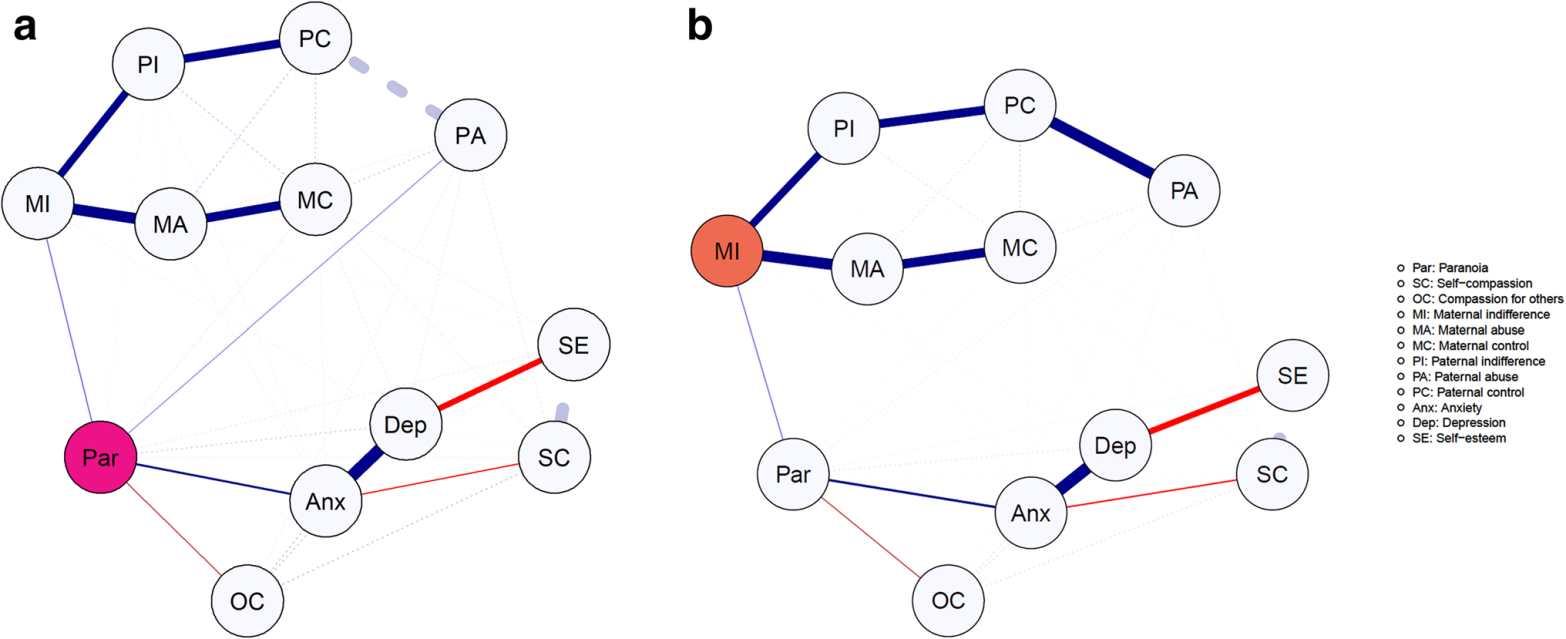
**a**, **b** Shortest path analysis

Figure 2a shows the shortest paths from paranoia to the other variables. The shortest path between paranoia and all parenting variables, except paternal abuse, was through maternal indifference, indicating that a large proportion of the relation between paranoia and the parenting variables is mediated by maternal indifference. Paternal abuse, however, retained its direct relationship with paranoia. Figure 2b shows the shortest paths from maternal indifference to all other variables. Together these figures show that the shortest paths to paranoia are separate for parenting behaviours and cognitive-affective variables.

## Discussion

This study presents the first investigation into potential links between specific parental behaviours—maternal and paternal overprotectiveness, abuse, and care—and paranoia. Associations were first analysed in a large epidemiological adolescent cohort, then replicated in a smaller non-epidemiological adult sample. The limitation of the brief measures used in the adolescent cohort was addressed by replicating associations in the adult participant group using stronger measures of the concepts, as well as adding several important cognitive-affective variables into the analysis. Finally, relationships were visualised in a network, enabling the strength of relationships and potential mediating pathways to be explored. All three levels of analysis revealed positive associations between paranoia and parental overprotection, indifference, and abuse, consistent with our first two hypotheses. It was found that the cognitive-affective variables were interacting with the parental behaviours and paranoia, but that within the network, cognitive-affective variables were not likely to be mediating the pathway between paranoia and parenting behaviour. Instead mediation is most likely to occur via maternal indifference, given the shortest path from paranoia to all parenting variables except paternal abuse was through maternal indifference.

Regressions using the single item measures of parenting and paranoia revealed consistent patterns across both participant groups. Increased levels of paranoia were associated with an increased likelihood of reporting parental verbal and physical abuse and lack of care. In the NSC-A group, there were also clear associations between the single item measure of increased parental over-protectiveness and paranoia, which was not replicated in the Oxfordshire participant group. It is not clear why this was the case. Analysis of the more extensive measures still showed a positive association between mother and father over-control and paranoia in this group. It is possible that the brief measure of over-protection did not satisfactorily capture the experiences of control measured in the MOPS. Correlations between the GPTS and MOPS showed similar levels of association with paranoia for all six subscales (maternal and paternal indifference, abuse, and control). Despite previous research finding stronger associations between reported paternal behaviour and schizophrenia, than maternal behaviour and schizophrenia [61], this was not found to be the case in our analysis regarding parenting and paranoia.

The network analysis revealed a highly interconnected network, though with the parenting and cognitive-affective variables clearly clustering separately. After controlling for all other variables, the strongest associations between reported parenting behaviours and paranoia were between maternal indifference and paranoia, and between paternal abuse and paranoia. Janssen et al. [20] suggest that adverse childhood experiences such as trauma or abuse may create cognitive vulnerability characterized by negative schemas about the self and the world, which then facilitates external attributions and the occurrence of paranoia. In line with this, we had hypothesised that cognitive-affective variables would provide a mediating pathway between parenting behaviour and paranoia. The associations between parenting behaviour and cognitive affective variables such as self-esteem, and between these variables and paranoia support this to an extent. However, the parenting variables and cognitive-affective variables had separate shortest paths to paranoia, suggesting that the variable clusters also have their own direct associations with paranoia. It is possible that other constructs not measured are mediators. For example, we did not measure attachment style because there are conceptual problems with the reliance on self-reported attachment style [24] and the interest was in more specific parenting behaviours. However, a measure of attachment style may have helped illuminate mediating pathways, by providing a measure of how individuals represent, internalize, and respond to their parents’ behaviours. Future research could examine where variables such as attachment style lie in the causal chain, along with other potentially relevant developmental variables such as family structure or sibling relationships.

There are a number of limitations to the study. First, demographic confounds such as socio-economic status and cognitive variables such as IQ were not tested. Previous studies assessing the relationship between early-life adversities and symptoms of psychosis have found that these variables are associated with paranoia [29]. True associations between parental behaviours and paranoia may be smaller once accounting for these factors. Second, although well validated, the measure of anxiety and depression was very brief. This was to minimise participant burden, particularly considering neither variable was of primary interest for this analysis. Nonetheless, edge strengths and mediating pathways concerning these variables may have been slightly altered had a more extensive measure been used. Third, there will be bias in the recruitment process for the adult Oxfordshire sample. Recruitment was achieved primarily through social media advertisements. Participants in this group were also predominantly female.

Finally, the biggest limitation is that the studies were cross-sectional, limiting causal inference. It cannot be determined whether these parental behaviours contribute to the development of paranoia, whether paranoia impacts parental relationships and thus parental behaviours, whether paranoia biases report of parental behaviours, or whether a confounding variable can explain the associations. However, regarding the possibility that paranoia biases the report of parental behaviours, it has been shown that patient reports of early experiences do tend to be unaffected by current symptoms, accurate when judged against reports of siblings, and stable over long periods, including times of acute illness versus remission [62–64]. Moreover, regarding the possibility that a confounding variable explains the relationship, a number of potentially confounding cognitive-affective variables were included in the analysis, yet were not mediating variables. On the other hand, there are a number of other variables that were not measured. For example, attachment style, bullying, and other victimisation experiences could be mediators. Additionally, although the two-item measure of anxiety included has shown sensitivity to identifying multiple anxiety disorders [50], other more in-depth or specific measures of may have revealed a mediating link that our measure did not capture.

Bradford Hill [65] argues that when judging whether effects might be causal, the strength and consistency of associations, temporal sequence of events, and the existence of plausible mechanisms should be considered. Upon these criteria, we argue a causal relationship between parenting and paranoia is certainly a possible explanation of the data, whereby parental abuse, indifference, and over-control could act as contributory causal factors in the development of paranoia. Further work testing this hypothesis is needed. For example, studies on longitudinal datasets would allow a greater degree of inference as to whether or not these links go beyond correlation, and studies in clinical populations would allow investigation of any association present in more severe cases of paranoia. This area is complex to research; there is reliance on retrospective reports and it is difficult to disentangle environmental and genetic contributions. However, there is a plausible mechanistic route that may be in action here.

## Data Availability

The NCS-A data can be accessed at https://www.hcp.med.harvard.edu/ncs/ncs_data.php. The Oxfordshire data can be made available upon appropriate request.

## References

[1] Freeman D, Garety PA, Bebbington PE (2005). Psychological investigation of the structure of paranoia in a non- clinical population. Br J Psychiatry.

[2] Bebbington PE, McBride O, Steel C (2013). The structure of paranoia in the general population. Br J Psychiatry.

[3] Wong KK, Freeman D, Hughes C (2014). Suspicious young minds: Paranoia and mistrust in 8- To 14-year-olds in the UK and Hong Kong. Br J Psychiatry.

[4] Freeman D (2016). Persecutory delusions: a cognitive perspective on understanding and treatment. Lancet Psychiatry.

[5] Zavos HMS, Freeman D, Haworth CMA (2014). Consistent etiology of severe, frequent psychotic experiences and milder, less frequent manifestations: A twin study of specific psychotic experiences in adolescence. JAMA Psychiatry.

[6] Fowler D, Freeman D, Smith B (2006). The Brief Core Schema Scales (BCSS): psychometric properties and associations with paranoia and grandiosity in non-clinical and psychosis samples. Psychol Med.

[7] Garety PA, Freeman D (2013). The past and future of delusions research: From the inexplicable to the treatable. Br J Psychiatry.

[8] Kesting ML, Lincoln TM (2013). The relevance of self-esteem and self-schemas to persecutory delusions: a systematic review. Compr Psychiatry.

[9] Tiernan B, Tracey R, Shannon C (2014). Paranoia and self-concepts in psychosis: a systematic review of the literature. Psychiatry Res.

[10] Freeman D, Evans N, Lister R (2014). Height, social comparison, and paranoia: An immersive virtual reality experimental study. Psychiatry Res.

[11] Atherton S, Antley A, Evans N (2016). Self-confidence and paranoia: an experimental study using an immersive virtual reality social situation. Behav Cogn Psychother.

[12] Freeman D, Pugh K, Dunn G (2014). An early Phase II randomised controlled trial testing the effect on persecutory delusions of using CBT to reduce negative cognitions about the self: The potential benefits of enhancing self confidence. Schizophr Res.

[13] Collett N, Pugh K, Waite F, Freeman D (2016). Negative cognitions about the self in patients with persecutory delusions: An empirical study of self-compassion, self-stigma, schematic beliefs, self-esteem, fear of madness, and suicidal ideation. Psychiatry Res.

[14] Kirby JN, Tellegen CL, Steindl SR (2017). A meta-analysis of compassion-based interventions: current state of knowledge and future directions. Behav Ther.

[15] Hickey T, Nelson B, Meadows G (2017). Application of a mindfulness and compassion-based approach to the at-risk mental state. Clin Psychol.

[16] Lincoln TM, Felicitas H, Hartmann M (2012). Can Paranoid Thoughts be Reduced by Targeting Negative Emotions and Self-Esteem? An Experimental Investigation of a Brief Compassion-Focused Intervention. Cognit Ther Res.

[17] Brown P, Waite F, Rovira A (2020). Virtual reality clinical-experimental tests of compassion treatment techniques to reduce paranoia. Sci Rep.

[18] Ascone L, Sundag J, Schlier B, Lincoln TM (2017). Feasibility and effects of a brief compassion-focused imagery intervention in psychotic patients with paranoid ideation: a Randomized Experimental Pilot Study. Clin Psychol Psychother.

[19] Read J, Agar K, Argyle N, Aderhold V (2003). Sexual and physical abuse during childhood and adulthood as predictors of hallucinations, delusions and thought disorder. Psychol Psychother Theory Res Pract.

[20] Janssen I, Krabbendam L, Bak M (2004). Childhood abuse as a risk factor for psychotic experiences. Acta Psychiatr Scand.

[21] Lieb R, Wittchen HU, Höfler M (2000). Parental psychopathology, parenting styles, and the risk of social phobia in offspring: A prospective-longitudinal community study. Arch Gen Psychiatry.

[22] Spokas M, Heimberg RG (2009). Overprotective parenting, social anxiety, and external locus of control: Cross-sectional and longitudinal relationships. Cognit Ther Res.

[23] Gotlib IH, Mount JH, Cordy NI, Whiffen VE (1988). Depression and perceptions of early parenting. Depress perceptions early Parent A. Longitud Investig.

[24] Read J, Gumley A (2008) Can Attachment Theory Help Explain the Relationship Between Childhood Adversity and Psychosis? In Ways towards secure attachment in family and society: International and Interdisciplinary Conference, Dec, 2007, Ludwig-Maximilians-Universitat, Munich, Germany

[25] Parker G, Tupling H, Brown LB (1979). A parental bonding instrument. Br J Med Psychol.

[26] Onstad S, Skre I, Torgersen S, Kringlen E (1994). Family interaction: parental representation in schizophrenic patients. Acta Psychiatr Scand.

[27] Pickering L, Simpson J, Bentall RP (2008). Insecure attachment predicts proneness to paranoia but not hallucinations. Pers Individ Dif.

[28] Lavin R, Bucci S, Varese F, Berry K (2020). The relationship between insecure attachment and paranoia in psychosis: A systematic literature review. Br J Clin Psychol.

[29] Bentall RP, Wickham S, Shevlin M, Varese F (2012). Do specific early-life adversities lead to specific symptoms of psychosis? a study from the 2007 the adult psychiatric morbidity survey. Schizophr Bull.

[30] Epskamp S, Borsboom D, Fried EI (2018). Estimating psychological networks and their accuracy: a tutorial paper. Behav Res Methods.

[31] Robinaugh DJ, Hoekstra RHA, Toner ER, Borsboom D (2019). The network approach to psychopathology: a review of the literature 2008–2018 and an agenda for future research. Psychol Med.

[32] Borsboom D, Cramer AOJ (2013). Network Analysis: An Integrative Approach to the Structure of Psychopathology. Annu Rev Clin Psychol.

[33] Epskamp S, Waldorp LJ, Mõttus R, Borsboom D (2018). The Gaussian graphical model in cross-sectional and time-series data. Multivar Behav Res.

[34] Borsboom D (2007). A network theory of mental disorders. World Psychiatry.

[35] Kessler RC, Avenevoli S, Costello EJ (2010). The National Comorbidity Survey Adolescent Supplement (NCS- A): II. Overview Des.

[36] Merikangas KR, Avenevoli S, Costello EJ (2009). National comorbidity survey replication adolescent supplement (NCS-A): I. background and measures. J Am Acad Child Adolesc Psychiatry.

[37] Sax LJ, Gilmartin SK, Bryant AN (2003). Assessing response rates and nonresponse bias in web and paper surveys. Res High Educ.

[38] Smith W (2008) Does Gender Influence Online Survey Participation? A Record-Linkage Analysis of University Faculty Online Survey Response Behavior. Online Submiss

[39] Waite F, Freeman D (2017). Body image and paranoia. Psychiatry Res.

[40] Green CEL, Freeman D, Kuipers E (2008). Measuring ideas of persecution and social reference: the Green et al. Paranoid Thought Scales (GPTS). Psychol Med.

[41] Statham V, Emerson LM, Rowse G (2019). A systematic review of self-report measures of paranoia. Psychol Assess.

[42] Freeman D, Antley A, Ehlers A (2014). The use of immersive virtual reality (VR) to predict the occurrence 6 months later of paranoid thinking and posttraumatic stress symptoms assessed by self-report and interviewer methods: a study of individuals who have been physically assaulted. Psychol Assess.

[43] Freeman D, Pugh K, Vorontsova N (2010). Testing the continuum of delusional beliefs: an experimental study using virtual reality. J Abnorm Psychol.

[44] Freeman D, Loe BS, Kingdon D (2019). The revised Green et al., Paranoid Thoughts Scale (R-GPTS): psychometric properties, severity ranges, and clinical cut-offs. Psychol Med.

[45] Parker G, Roussos J, Hadzi-Pavlovic D (1997). The development of a refined measure of dysfunctional parenting and assessment of its relevance in patients with affective disorders. Psychol Med.

[46] Raes F, Pommier E, Neff KD, Van Gucht D, Raes F, Pommier E, Neff KD, Van Gucht D (2011). Construction and factorial validation of a short form of the Self-Compassion Scale. Clin Psychol Psychother.

[47] Pommier EA (2010). The compassion scale. Diss Abstr Int Sect A Humanit Soc Sci.

[48] Kroenke K, Spitzer RL, Williams JBW, Lowe B (2009). An ultra-brief screening scale for anxiety and depression: the PHQ-4. Psychosomatics.

[49] Löwe B, Wahl I, Rose M (2010). A 4-item measure of depression and anxiety: Validation and standardization of the Patient Health Questionnaire-4 (PHQ-4) in the general population. J Affect Disord.

[50] Kroenke K, Spitzer R, Williams J (2007). Anxiety disorders in primary care: prevalence, impairment, comorbidity, and detection. Ann Intern Med.

[51] Rosenberg M (1965). Society and the Adolescent Self-Image.

[52] IBM (2016) SPSS, version 24

[53] Kessler RC, Ph D, Avenevoli S, et al (2010) Design and Field Procedures in the US National Comorbidity Survey Replication Adolescent Supplement (NCS-A) Ronald. 18:69–83. 10.1002/mpr.279.Design 10.1002/mpr.279 PMC2774712 19507169

[54] Maxwell SE, Cole DA (2007). Bias in cross-sectional analyses of longitudinal mediation. Psychol Methods.

[55] R Core Team (2013) R, version 3.6.1

[56] Epskamp S, Cramer AOJ, Waldorp LJ (2012). Qgraph: Network visualizations of relationships in psychometric data. J Stat Softw.

[57] Tibshirani R (1996). Regression shrinkage and selection via the Lasso. J R Stat Soc.

[58] Epskamp S, Fried EI (2018). A tutorial on regularized partial correlation networks. Psychol Methods.

[59] Foygel R, Drton M (2010) Extended Bayesian information criteria for Gaussian graphical models. In: Adv Neural Inf Process Syst 23 24th Annu Conf Neural Inf Process Syst 2010, NIPS 2010, pp 1–14

[60] Dijkstra EW (1959). A note on two problems in connexion with graphs. Numer Mathermatik.

[61] Read J, Gumley A (2008). Can attachment theory help explain the relationship between childhood adversity and psychosis?. New Dir Psychother Relational Psychoanal.

[62] Fisher HL, Craig TK, Fearon P (2011). Reliability and comparability of psychosis patients’ retrospective reports of childhood abuse. Schizophr Bull.

[63] Parker G, Fairley M, Greenwood J (1982). Parental representations of schizophrenics and their association with onset and course of schizophrenia. Br J Psychiatry.

[64] Rankin P, Bentall R, Hill J, Kinderman P (2005). Perceived relationships with parents and paranoid delusions: comparisons of currently ill, remitted and normal participants. Psychopathology.

[65] Bradford Hill A (1996). The environment and disease: association or causation?. Proc R Soc Med.

